# Are humans constantly but subconsciously smelling themselves?

**DOI:** 10.1098/rstb.2019.0372

**Published:** 2020-04-20

**Authors:** Ofer Perl, Eva Mishor, Aharon Ravia, Inbal Ravreby, Noam Sobel

**Affiliations:** Department of Neurobiology, Weizmann Institute of Science, Rehovot 76100, Israel

**Keywords:** body-odour, face-touching, self-sampling, self-sniffing, social chemosignalling

## Abstract

All primates, including humans, engage in self-face-touching at very high frequency. The functional purpose or antecedents of this behaviour remain unclear. In this *hybrid review*, we put forth the hypothesis that self-face-touching subserves self-smelling. We first review data implying that humans touch their faces at very high frequency. We then detail evidence from the one study that implicated an olfactory origin for this behaviour: This evidence consists of significantly increased nasal inhalation concurrent with self-face-touching, and predictable increases or decreases in self-face-touching as a function of subliminal odourant tainting. Although we speculate that self-smelling through self-face-touching is largely an unconscious act, we note that in addition, humans also consciously smell themselves at high frequency. To verify this added statement, we administered an online self-report questionnaire. Upon being asked, approximately 94% of approximately 400 respondents acknowledged engaging in smelling themselves. Paradoxically, we observe that although this very prevalent behaviour of self-smelling is of concern to individuals, especially to parents of children overtly exhibiting self-smelling, the behaviour has nearly no traction in the medical or psychological literature. We suggest psychological and cultural explanations for this paradox, and end in suggesting that human self-smelling become a formal topic of investigation in the study of human social olfaction.

This article is part of the Theo Murphy meeting issue ‘Olfactory communication in humans’.

It's not what you look at that matters, it's what you see

     —Henry David Thoreau

## 1. Face-touching may reflect unconscious hand-smelling

Mammalian smelling largely depends on sniffing [1–5]. This is clearly evident in the stereotypical terrestrial mammalian behaviour of olfactory self-investigation: we look at rodents, canines and felines, and see animals that are often sniffing themselves or their own bodily secretions. Why don't we see this when we look at humans? The lay person may answer that: ‘well, humans just don't smell themselves like mice, cats and dogs do’. However, to paraphrase the opening Thoreau quote, we think this lay answer reflects what people choose to see, and if we looked at human behaviour differently, we would in fact see an animal that is often sniffing itself. The primary hypothesis that we put forth in this hybrid review manuscript is that the very prevalent human behaviour of face-touching in fact often subserves hand-smelling, and hence, social olfaction.

## 2. Face-touching in primates

Face-touching is a prevalent primate behaviour. In 20 min observations, gorillas, chimpanzees and orangutans touched their own faces an average of 19.87, 24.2 and 12.12 times respectively, i.e. about once a minute [6]. Moreover, these face-touches were predominantly with the left hand [6]. Whereas the above study failed to observe a similar extent of face-touching in monkeys, a later study found 51.33 face-touches per hour in monkeys as well, i.e. also about once a minute [7]. This later study did not observe laterality in the monkey behaviour, and also argued against the claim on laterality in apes [7]. Regardless of lateralization, primates clearly touch their own faces at high frequency, and this behaviour has been labelled as puzzling [6]. It has been labelled as such because its function remains unknown. A major hypothesis of this hybrid review article is that the function of face-touching is hand-smelling. We put forth the hypothesis that this is a path to obtaining olfactory information on self and on others.

## 3. Face-touching in humans

The above-quoted study that observed primates also observed humans: over a 20 min observation, 18 participants touched their faces an average of 13.33 times, i.e. at a rate similar to orangutans [6]. It was unclear whether participants in this study knew they were being observed, or why. In turn, because of its role as a possible path for disease propagation, face-touching has been incidentally quantified in the study of infectious disease. For example, one study reports on 10 participants (5 women), each video-observed individually for a period of 3 h seated at a desk performing ‘office tasks’ [8]. The participants knew they were being filmed for later behavioural analysis. Even though this knowledge may have increased self-awareness and minimized self-touching, they nevertheless brought their own hand to their faces approximately 16 times per hour [8]. Moreover, approximately five touches per hour were directed specifically at the nostrils. A second study reports on 26 participants, medical students who were videoed while attending two 2 h lectures (i.e. 4 h of group observation) [9]. These participants also knew they were being filmed for later behavioural analysis, but did not know the question being addressed. In this study, face-touching occurred approximately 23 times per hour, and approximately seven touches per hour were directed specifically at the nostrils [9]. In a third study, 79 family-medicine doctors and staff were observed within a clinical setting. Although they knew they were being observed, they did not know that the question of interest was face-touching. Despite iteration of proper contact hygiene in this population, they nevertheless touched their own eyes, nose and mouth approximately 10 times an hour across a 2 h observation [10]. We note that this behaviour of face-touching may be responsible for transferring nearly 25% of respiratory illness [11]. Given this potentially significant contribution to disease, simple evolutionary thinking implies there must also be very meaningful advantages to self-face-touching; otherwise, this behaviour would have been minimized in the human behavioural repertoire in light of its price.

## 4. Is face-touching an olfactory behaviour?

**Figure 1.**
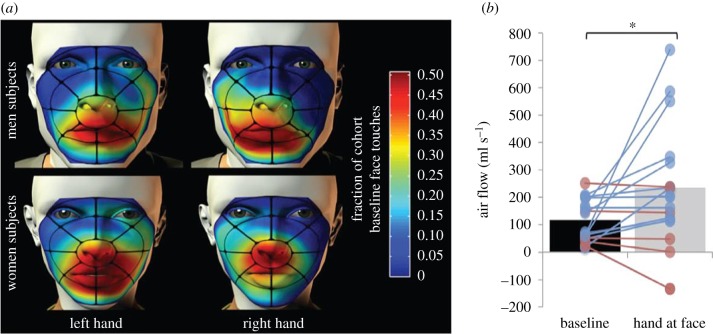
Humans often bring a hand to their nose and sniff it. (*a*) A heat-map of face-touching in 160 participants. Colour reflects the proportion of the cohort that increased face-touches over baseline at that facial location (red = increase). Most face-touching is directly at or around the nose. (*b*) Measurement of nasal airflow during face-touching versus non-face-touching periods in the same session in 17 participants. Blue lines reflect participants that increased airflow and pink lines reflect participants that decreased airflow. Face-touching was accompanied by near doubling of nasal airflow, or in other words, a sniff. Adapted from [12]. (Online version in colour.)

This question was only addressed in one study, conducted in our laboratory [12]. In this study, we used video to observe 160 participants, each seated alone in the observation room, unaware of the observation process or interest. Participants were observed for 3 min before and 3 min after an experimenter (either same or opposite sex) entered the experimental room to greet them either with or without a handshake. We defined a face-touch for possible hand-sniffing as contact application of the hand to the faces, below the eyebrows, and above the lower lip. The use of video allowed us to not only count face-touches, but also time them. We observed that participants seated alone in a room (before a handshake took place) brought a hand into the vicinity of their nose and kept it there for approximately 22% of the time [12] (figure 1*a*).

**Figure 2.**
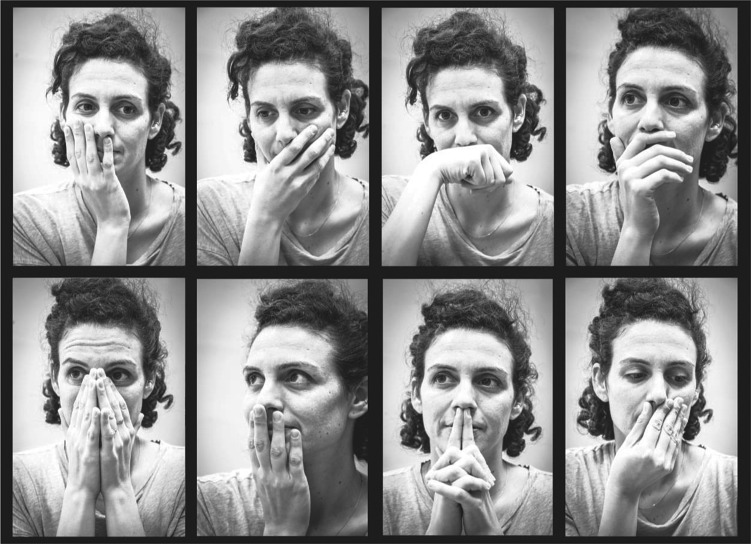
Illustration of typical postures that may serve subconscious hand-smelling. We reviewed the several hundred participants in our videoed experiments and identified numerous stereotypical postures for placing the hands at the nose. Given that these were often accompanied by a measurable sniff, we propose that these postures subserve subconscious hand-sniffing. This is a staged illustration figure, and the presented postures reflect a typical but not exhaustive set. (See also figure 10*a*.)

In other words, humans are bringing their hands to their nose, and keeping them there, at an astonishingly high rate. But are they sniffing these hands? Perhaps this is all merely face-touching as a form of displacement behaviour. Human displacement behaviour is pronounced, and indeed increased in moments of stress, or in pathological conditions associated with anxiety [13]. To address this important alternative, in a separate control study with 33 participants, we measured nasal airflow during such hand-to-face encounters. We typically define a sniff as a greater than 15% change in normalized sniff volume from baseline respiration (increase (as often after pleasant odours) or decrease (as often after unpleasant odours)), and/or a modulation in sniff volume reflecting a shift in standard deviation (s.d.) > 0.35 over ongoing respiration [14]. We found that nasal airflow more than doubled when the hand was at the nose (12 of 17 participants increased (binomial probability *p* = 0.047), group baseline flow = 112.75 ± 75.56 ml s^−1^, hand-at-face flow = 237.81 ± 220.82 ml s^−1^, *t*_16_ = 2.37, *p* = 0.03) [12], or in other words, people are sniffing their own hands (figure 1*b*). Moreover, in a second control described later in more detail, we covertly scented the experimental environment. This significantly increased or decreased the rate of face-touching in a predictable odourant-specific manner (figure 9*a*). These two control studies suggest to us that the very prevalent behaviour of face-touching is driven in part by olfaction. We identified several ‘typical’ postures of what we think is hand-sniffing that consistently reoccurred across participants (figures 2 and 10*a*). Somehow, we are all looking at this behaviour, but mostly not seeing it.

**Figure 3.**
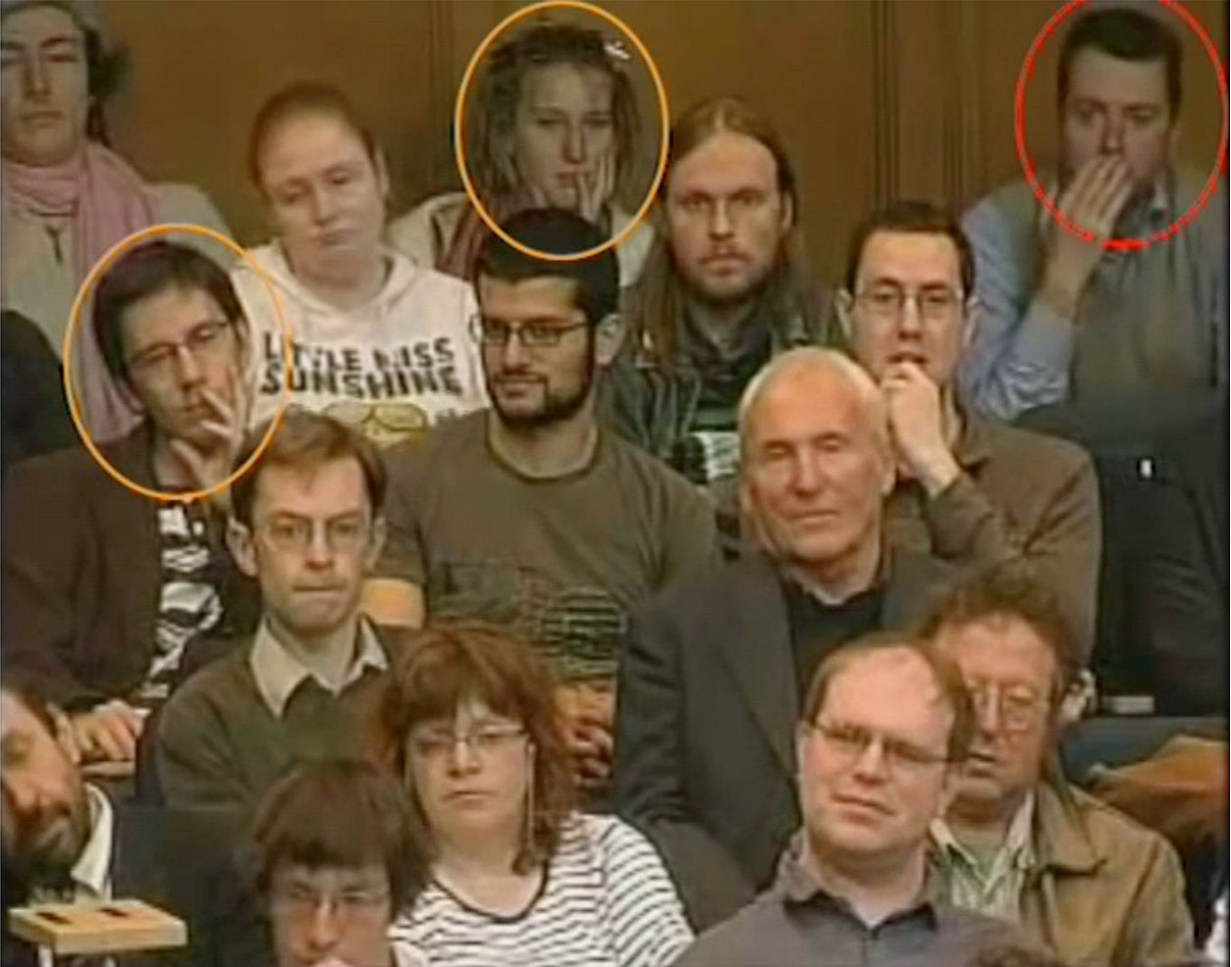
Humans who may be sniffing their hands. A screen-shot from electronic supplementary material, video S1 ( also available at https://youtu.be/mKpOGdIVjDI), time: 00:40. In the edited video, we used orange circles to highlight individuals with a hand at their nose, and red circles to highlight those cases where you can literally ‘see’ the action of sniffing (although we think hand-sniffing is occurring in almost all of these cases, albeit less explicitly). The person to the lower left of the individual indicated with the red circle is not highlighted because his hand is not above his lower lip, thus not meeting our criteria for hand-sniffing face-touches. We further turn the reader’s attention to time point 00:52 in electronic supplementary material, video S1 for our favourite instance in this edited video, where despite the lack of any information (it is soundless), it appears that the red-highlighted person is self-consciously embarrassed by something that was said and appears to sniff her own hand in what we speculate might be reassurance. (Online version in colour.)

We invite the reader to engage in the following observational experiment: next time you sit at a seminar or an online video meeting, take a moment to look not at the speaker, but rather at your fellow audience. How many people have their hands at their nose? Typically, you will observe that over time this accounts for a large proportion of the audience. Moreover, look carefully, and you will often see the unmistakable action of sniffing. To further illustrate this, we mined YouTube for a seminar audience video and invite the reader to look at the only one we found (original full-length version: https://www.youtube.com/watch?v=I_dpPc-XQ6w). In our view, it is quite remarkable (electronic supplementary material, video S1, also available at https://youtu.be/mKpOGdIVjDI) (figure 3). We stress, however, that here this is our interpretation of the behaviour, because unlike in the above-reported experiments where we verified that hand-to-face touching was accompanied by sniffing and modified by odourants, here this merely reflects our speculation.

## 5. When humans sniff their hands, they are also smelling where their hands touched

When people sniff their own hands, they are not smelling their intrinsic odour alone. First, they may also be smelling added fragrance such as perfume. Remarkably, such perfumes may be specifically self-selected so as to amplify the olfactory-related signature of one's own human leukocyte antigen (HLA) [15]. Moreover, they are also smelling where their hands were previously. A common, if not formally quantified behaviour, is placing the hand at an erogenous body location, and then sniffing it. This behaviour is often seen in public in toddlers or small children who have yet to internalize the associated social taboos. The behaviour likely persists in adults, albeit mostly in private. The reason we describe this behaviour as common is because of its Web presence: a simple Google search for [‘hand sniffing’ in children] yields approximately 6500 results. A large number of these are parents describing what they fear is excessive hand-sniffing in their children. In contrast with this prevalence implicated by the common Web search, the phenomenon of olfactory self-sniffing has only minimal mention in the medical or psychological literature. For example, the exact same search that yielded approximately 6500 results in Google yielded zero results in PubMed and PsycNet/PsychInfo. We are hard-pressed to think of a human behaviour that is so wide-spread, that troubles so many people, that clearly reflects underlying processes with developmental, clinical and social relevance, and yet has so little traction in the formal medical/psychological record.

To gain a further sense of prevalence of these and associated behaviours, for the purpose of this review, we circulated a brief self-report questionnaire (electronic supplementary material, file S1) (interested readers can still participate at https://forms.gle/A5JK5Aq4LpmM1Gtb7).

## 6. Questionnaire methods

The questionnaire probed for age, gender and country of residence and then went on to ask 11 questions concerning smelling of self and significant others, and one question on behaviour of offspring. Participants reported the prevalence of olfactory behaviours (never/rarely/occasionally/often) and could skip any question. In cases where more than one answer was given for a question, we discarded that entry. There were 33 such deletions, amounting to less than 1% of the data. Whereas most of the report of this effort is descriptive, sex-differences were estimated using a Cramér–von Mises (CvM) test [16]. The CvM test compares the cumulative density functions of two distributions and is thus suitable for ordinal variables. The *p*-value for this statistic was estimated using bootstrap analysis. The group labels were randomly assigned to the ratings 1 000 000 times, and a CvM statistic was calculated for each assignment. By that, a relevant CvM statistic distribution was generated and was used to estimate the *p*-value. Critical alpha was set at *p* < 0.05. We finally corrected the *p*-values for the 10 questions using Benjamini–Hochberg false discovery rate (FDR) correction. We acknowledge that this effort suffers from all the limitations associated with self-report: the sampled population is biased (we circulated through our own social media paths), answers are a reflection of self-perceived behaviour rather than behaviour itself, and so on [17]. These limitations are here further accentuated given that we are dealing with a behaviour that is largely subconscious, and often treated as taboo [18]. Nevertheless, this effort sheds initial light on behaviours that otherwise have almost no reflection in the literature.

## 7. Questionnaire results

**Figure 4.**
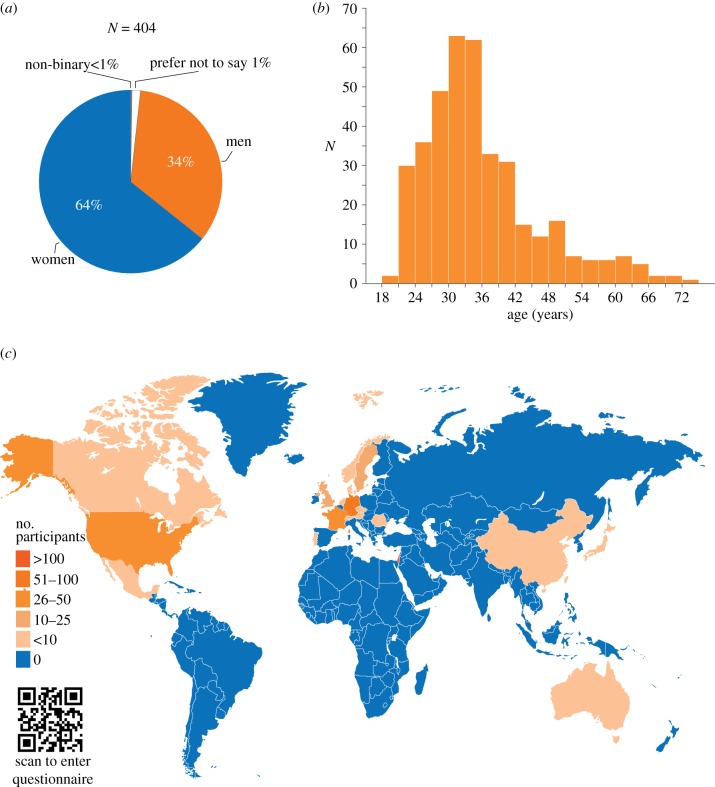
Online self-report survey demographics. (*a*) Respondent proportions by sex. (*b*) Respondent proportions by age. (*c*) Respondent geographical distribution. Map by Freepik. (Online version in colour.)

We obtained 404 responses from 137 men and 260 women (figure 4*a*), ranging in age from 19 to 74, mean age 35.28 ± 10.39 years old (figure 4*b*), and who live in 19 different countries (figure 4*c*) (the complete raw data are available in electronic supplementary material, file S2). We excluded data from five participants; one was underage, and four had five or more uninformative answers (n.a. or blank). Seven respondents did not report gender and were thus unavailable for the gender comparisons.

**Figure 5.**
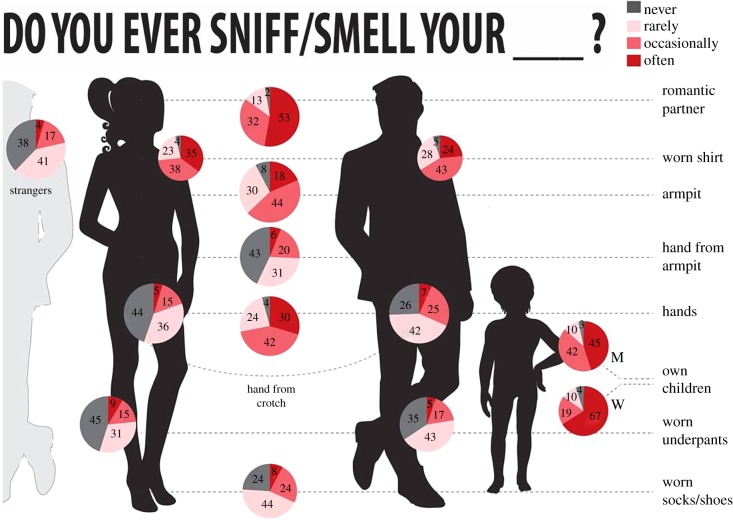
Humans self-report sniffing themselves and their conspecifics. Distribution of replies to all the questions asked in the questionnaire. The values reflect percentage (rounded) of respondents. M = men, W = women. For questions with a significant sex-difference in replies, each sex is represented separately. (Online version in colour.)

These participants provided self-report on various olfactory sampling activities, which they rated as engaging in either never, rarely, occasionally or often (detailed in infographic figure 5). Whereas 60.64% of respondents reported sniffing/smelling strangers, an overwhelming 94.34% of individuals reported sniffing/smelling their close relations. Men and women reported sniffing their romantic partners equally, but women reported sniffing their children more than did men (sniffing often: women = 66.67%, men = 45.00%, CvM = 0.80, bootstrap *p* = 0.025, corrected *p* = 0.125). A similarly overwhelming 94.31 and 91.58% of respondents reported sniffing/smelling their own hands and armpits, respectively. As to placing hands in either the armpit or crotch, and then sniffing them, while 55.94% of all respondents reported doing this with the armpits, 73.89% of men and 55.69% of women reported doing this with the crotch (CvM = 1.41, bootstrap *p* = 0.0002, corrected *p* = 0.002). Finally, respondents also reported high rates of sniffing clothes that they had worn. All of these results are detailed in the infographic figure 5.

**Figure 6.**
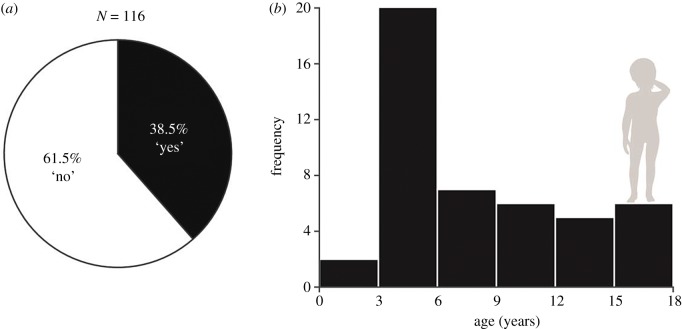
Parents report increased hand-sniffing in children aged 3–6. (*a*) Nearly 40% of parents noticed a stage of increased hand-sniffing in their children. (*b*) The frequency of reported hand-sniffing in children by age. An increase was observed primarily between the ages of 3 and 6.

Finally, 182 respondents (60 M) were parents. Whereas 61.54% of parents reported no awareness of a phase of increased hand-sniffing in their children, 38.46% did report noticing such a period, which peaked between the ages of three and six. Confirming that nearly 40% of parents observe such a phase may here serve to mitigate the concerns of those parents who think their child's behaviour is aberrant in this respect (figure 6).

**Figure 7.**
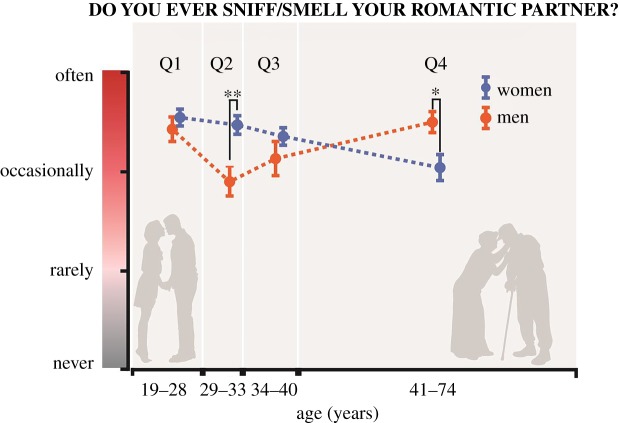
Self-reported romantic partner-sniffing as a function of age. Whereas in the age-group 29–33, women reported significantly higher partner-sniffing than men, this pattern shifted such that in the age-group 41–74, men reported more partner-sniffing than women. Q1 *n*_F_ = 9, *n*_M_ = 30; Q2 *n*_F_ = 72, *n*_M_ = 30; Q3 *n*_F_ = 61, *n*_M_ = 29, Q4 *n*_F_ = 48, *n*_M_ = 40. We have no explanation for this pattern.

We further investigated whether there were questions that were rated differently with age. To this end, we grouped respondents into four equally sized age quartiles (ages 19–28, 29–33, 34–40 and 41–74, denoted here by Q1, Q2, Q3 and Q4, respectively) and asked whether rating distributions for specific questions significantly varied across these age groups, both overall and within-sex. We found significant differences for one question only: ‘Do you ever sniff/smell your romantic partner?', both within each sex and overall. For women, three out of six comparisons were significant, and for men two out of six comparisons were significant (women Q1 to Q4 CvM = 1.69, bootstrap *p* = 0.0007, corrected *p* = 0.0057; women Q2 to Q4 CvM = 1.15, bootstrap *p* = 0.0057, corrected *p* = 0.023; women Q1 to Q3 CvM = 0.71, bootstrap *p* = 0.0317, corrected *p* = 0.0846; men Q2 to Q4 CvM = 1.31, bootstrap *p* = 0.0022, corrected *p* = 0.012; men Q1 to Q2 CvM = 0.64, bootstrap *p* = 0.038, corrected *p* = 0.087) (figure 7). We finally asked whether there were differences between men and women for each age group and found that for Q2 women sniffed their romantic partners more than men (CvM = 1.75, bootstrap *p* = 0.0021, corrected *p* = 0.002) and for Q4 men sniffed their partners more than women (CvM = 0.83, bootstrap *p* = 0.016, corrected *p* = 0.016) (figure 7). This is a rather striking dissociation for which we do not have a good explanation.

We conclude from all this that although in our view most of olfactory social sniffing is subconscious, upon being asked, humans are nevertheless quite aware of engaging in this behaviour.

## 8. When humans sniff their hands, they are also smelling who their hands touched

**Figure 8.**
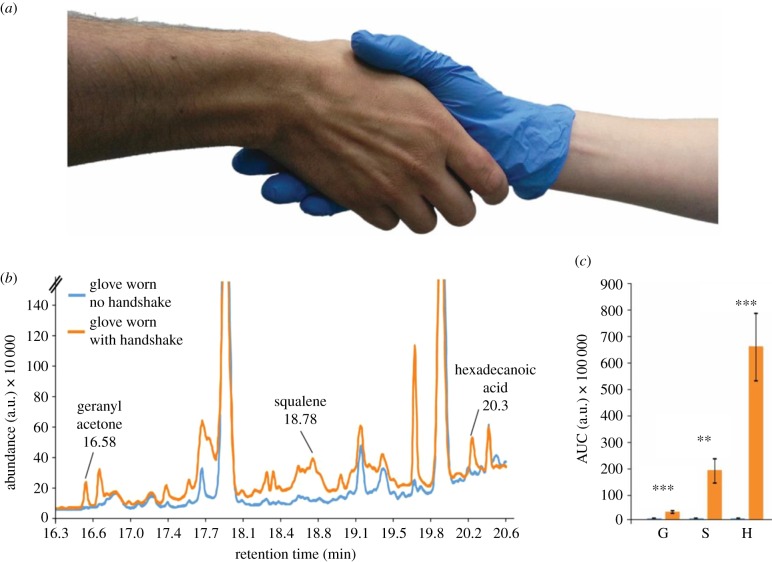
Handshaking transfers volatiles from skin. (*a*) A representative image of our sampling method using a nitrile glove during handshake. (*b*) An example chromatogram from one experiment. Note that the ‘clean' condition is a glove worn by the same hand, but not shaken. The only three peaks that were present following all shakes but never once in the control are those we describe in the following panel. (*c*) Summated data from 10 individuals (each an average of three shakes) demonstrating three compounds of interest in chemosignalling (geranyl acetone (G), squalene (S) and hexadecanoic acid (H)) that were effectively transferred by handshaking in all instances and never once in the control. Error bars show standard error, **p < 0.01, ***p < 0.001. a.u., arbitrary units. Adapted from [12]. (Online version in colour.)

After observing the extent of human hand-sniffing, it occurred to us that this behaviour may provide information not only regarding one's own body, but also regarding the bodies of others. Indeed, in the previously described extensive hand-sniffing in primates (figure 10*c*), the animals typically mix between merely sniffing their own hands spontaneously, or sticking their fingers within bodily orifices (often the nostrils) of conspecifics and then carefully sniffing their own hand [19]. Humans obviously touch each other within the context of close relationships, but they also touch complete strangers: handshaking is a common introductory greeting ritual in the West and beyond [20,21]. We therefore set out to test in our previous study [12] the hypothesis that handshaking subserves social chemosignalling. We first used gas-chromatography mass-spectrometry (GCMS) to test whether the brief contact of a handshake is sufficient to transfer volatile organic compounds from one hand to another and found evidence for extensive transfer of several molecules from skin (figure 8).

**Figure 9.**
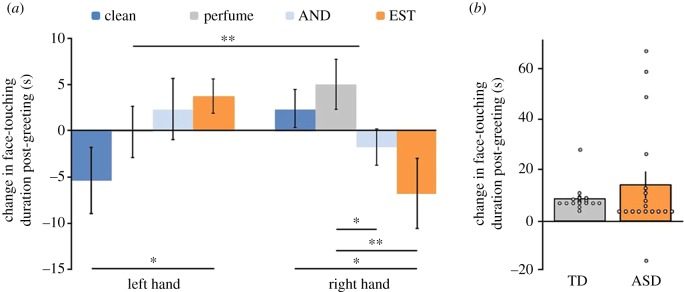
Humans sniff their hands after handshaking. (*a*) Change in face-touching duration post-handshake within-sex in 66 women. Different conditions were either untainted, or tainted with odour (either a generic perfume, or one of the steroidal compounds 4,16-androstadien-3-one (AND) or oestra-1,3,5(10),16-tetraen-3-ol (EST)). Sniffing of the shaking hand (right) increased after handshake, increased even more in the presence of a perfume, but reversed in the presence of the steroids. A mirror-image pattern is evident in the left hand. *p < 0.05, **p < 0.01. Adapted from [12]. (*b*) Change in face-touching duration post-handshake within-sex in 18 men with autism (ASD) and 18 typically developed men (TD). Adapted from [22]. (Online version in colour.)

We then covertly recorded and quantified hand-to-nose contact before and after handshaking with an experimenter within a fixed paradigm. What we view as the most significant result of this effort was the astonishingly high baseline rate of hand-sniffing, as reported above (figure 1). However, we also observed that this behaviour was significantly impacted by handshaking. Participants significantly increased sniffing of the shaking hand (right) after within-sex handshakes, yet increased sniffing of the non-shaking hand (left) after cross-sex handshakes. In other words, handshaking impacts ensuing hand-sniffing. Moreover, to again verify the olfactory origins of this behaviour, in a separate control experiment with 63 participants, we artificially tainted the body-odour of the experimenter. Here the experimenter wore a device on his/her wrist that covertly emitted one of three odourants during handshake. We found that we could selectively increase or decrease hand-sniffing in participants as a function of the odourant we used to taint the experimenter, and all this despite using subliminal levels of odour (figure 9*a*). This further verifies the olfactory origins of this behaviour and, in our view, serves not only to mitigate, but potentially to reverse our initial concern regarding this merely being a reflection of displacement behaviour. In using the term ‘reverse’, we mean that not only do we think that the behaviour we observed is chemosignalling and not displacement behaviour, but moreover, we suspect that many of the previous reports of displacement behaviour may in fact reflect chemosignalling. As to the counterintuitive within-sex versus cross-sex dissociation, it was very powerful (ANOVA, three-way interaction: *F*_1,77_ = 37.79, *p* < 0.0001), yet we have no strong theoretical hypothesis for it. We speculate that whereas sniffing the shaking hand provides information about the conspecific in question, sniffing the non-shaking hand provides comparative information on *self* (more about this in §9). We do not know, however, why one would want to increase this type of information on same-sex conspecifics rather than on cross-sex conspecifics and can only speculate that the sex interaction may reflect context specificity: although participants increased sniffing the within-sex shaking hand in the context of our experiment, they may opt to increase sniffing cross-sex hands in other behavioural settings. This remains a question for investigation.

Amusingly, the handshake effect has been ‘replicated’ in live recreations on several popularized science television shows around the world (electronic supplementary material, figure S1 and video S2), and we have now replicated the handshake effect in one additional published study. Given our overarching working hypothesis that social chemosignalling is a big part of human social interaction, we therefore hypothesized that social chemosignalling will be altered in disorders of social behaviour. A particularly relevant condition is autism spectrum disorder (ASD). With this in mind, we replicated the handshake paradigm in 18 cognitively able adult men with ASD [22]. The effect replicated in the ASD cohort as a group, and moreover, four members of the ASD cohort stood out with hand-sniffing behaviour that was inordinately persistent and explicit (figure 9*b*). After handshake, three of the four practically did not remove their hand from their nose for the duration of the recorded session. Their behaviour reflected explicit careful olfactory investigation. In turn, the fourth participant carefully sniffed his hand for much of the baseline, but then never brought it back to his nose after handshake. In other words, consistent with other measures [23], the variability in the ASD cohort was higher than in controls. In conclusion, this result in ASD replicates the effect of increased face-touching after handshake, but the limited size of the cohort prevents us from determining whether this increase is greater in ASD than in controls, despite a trend to that effect. This dovetails with reports on excessive olfactory social investigation in ASD. In fact, the observation that initially drove us to investigate social olfaction in ASD in the first place was from a newspaper weekend magazine story in the leading Israeli newspaper *Haaretz*. This was the story of Ayala, a 51-year-old woman, who was one of the first in Israel to be diagnosed with autism (the English translation of the story is currently viewable at: http://www.haaretz.com/weekend/magazine/eternal-child-1.342756). Ayala is non-verbal, and her primary avenue of social investigation always was, and still is, her sense of smell. Ayala uses her nose to investigate all individuals. The following is a quote from the story:

When Ayala was four, her mother took her to preschool and stayed with her there. ‘I thought that if she heard children talking she'd pick something up’, she explains. ‘The children really loved her, but she didn't cooperate with them. The most important thing to her, even then, was smell. She would smell the children. If she liked the smell, you were her friend. If not, you couldn't come near her’.

This overt pattern of olfactory social investigation, or social sniffing, is repeated often in more detailed case-report depictions of behaviour in ASD (e.g. Stephen Wiltshire in [24]).

## 9. Why do humans smell themselves?

**Figure 10.**
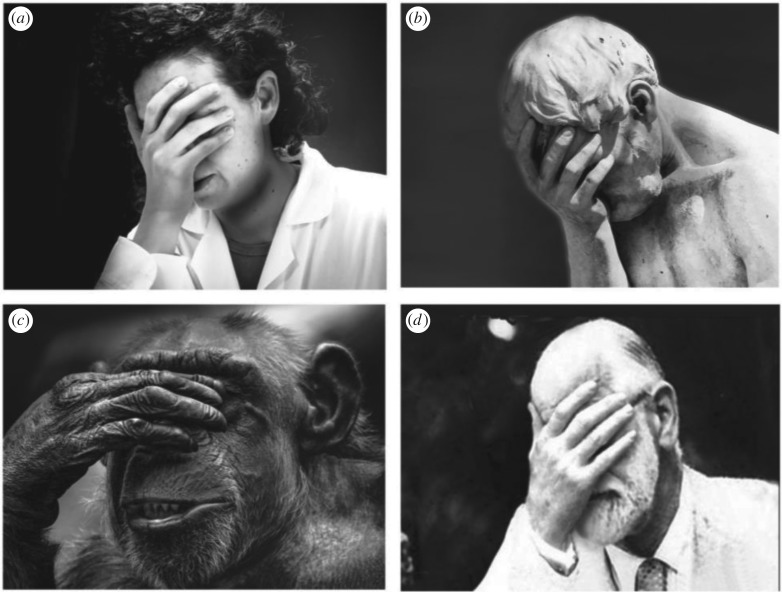
It's not what you look at that matters, it's what you see. (*a*) An additional typical posture of placing the hand at the nose. (*b*) The same typical posture, here in Henri Vidal's 1896 sculpture of Cain after killing his brother Abel. (*c*) The same typical posture, here in a non-human primate. (*d*) The same typical posture, here witnessed in Sigmund Freud himself (1935). Although others may see in these images displacement behaviours, what we see in all four images are apes smelling the inside of their hand.

As implied from the above, humans sniff their hands in part in order to obtain information on others whom they've touched, information that may be processed in dedicated brain networks [25–27] to provide assorted and important information on conspecifics (the types of information shared through human chemosignalling have recently been reviewed in [28–30]). However, given the potentially high frequency of human olfactory self-sniffing (e.g. approx. 20 times per hour), and its persistence in isolation, it is more likely that this behaviour primarily provides information on intrinsic and not external sources. So why do people smell themselves? In answering this question, we first make an important distinction between conscious versus subconscious olfactory self-investigation. To get a sense of this, we can similarly ask why do people consciously look at themselves in mirrors? Most people will probably answer that it is in order to verify that they ‘look good’. Similarly, people may consciously sniff themselves in order to verify their own odour, although paradoxically, most people will note that it is to verify that they ‘don't smell bad’. This distinction between wanting to ‘look good’ on one side, and ‘not smell bad’ on the other, may reflect fears related to the morality of malodour [31], an issue we will return to later in this article. Moreover, people may also consciously sniff themselves in order to detect signs of disease [32]. These reasons for conscious olfactory self-sampling, interesting though they may be, are not at the core of what we think is important in this behaviour. It is the incessant subconscious action of olfactory self-sampling that we think tells a more important story. To stick with the visual mirror analogy, parents intuitively know to imitate or mirror their developing child's sounds and actions [33]. This mirroring provides the developing infant with the information that he/she is a separate independent being that can trigger reactions in others and this gives rise to a sense of *self* [33]. Similar reflection and reassurance of *self* is provided by a glass mirror as well, and this may be one of the inherent incentives for its use [34]. Given that mirrors have not been around since the dawn of humanity [35], a sense of *self* can likely be formed without one at hand. We propose that the path by which humans could have always observed themselves to get a notion of *self* is by olfaction. Thus, we think that in sniffing our own body, we are subconsciously obtaining an external reflection and reassurance of *self*. This is consistent with the increased face-touching in times of stress, previously viewed as displacement behaviour. Just as looking in the mirror can serve to reduce stress and anxiety by reassuring a sense of *self* [36], so can smelling oneself. It is through this prism that we view most of face-touching behaviour. For example, humans are said to hide their faces in their hand when they sense shame. But why do they do this? We argue that sniffing the inside of the hand provides a reassuring signal of *self* that aids in managing such *self*-threatening emotions (figure 10, electronic supplementary material, video S1, time 00:52). One may argue that humans are not that good at recognizing their own body-odour [37–39]. However, several studies have suggested that humans can in fact recognize their own smell [39–43], and that this ability is evident even in children [44]. Moreover, implicit measures typically uncover self-recognition of body-odour in the very same individuals who could not explicitly identify their own smell [37,39]. This dissociation may imply that olfaction subserves mostly unconscious contributions to a sense of *self*, or what has been referred to as a ‘*minimal self*’ [45].

Mirror behaviour also serves to illustrate a second and critical role we see for olfactory self-sniffing. This illustration arises from a somewhat surprising outcome of a behaviour termed *mirror-fasting*, namely the self-imposed avoidance of looking in the mirror. Although we could not find any formal investigations of this relatively recent behavioural fad, it does have representation online, and in one informal review, it was noted that a rather universal outcome of mirror-fasting is ensuing avoidance of social interaction [46]. For example, in a report on one week of mirror-fasting [47], the fourth of 11 reported effects was as follows:

4. I purposely avoided people.

I felt insecure, shy, and I wanted nothing more than to just avoid people and avoid wondering what was crossing their minds as they looked at my face. As I mentioned, when I did speak with others, it was a lot of me playing with my hair and staring at my feet. So I began to just avoid seeing people altogether. It made things easier, and I felt more secure.

Whereas reduced social interaction is to us a surprising outcome of glass mirror-fasting, a role of olfactory self-sampling in social interaction is in our view highly likely. Many social chemosignalling behaviours rely on comparing self-odour with others. For example, humans may optimize mate selection based on genetic compatibility (particularly in the HLA complex) [48], and this can be inferred from body-odour [49,50]. This process, by definition, entails a comparison to self-body-odours. Moreover, we speculate that this holds true for dyadic interactions beyond those directed at mating alone. For example, close friends have more similar genetics than expected by chance [51,52]. Assuming friends do not sequence each other before deciding on their friendship, this implies that they must compare some traits that inform on genetic makeup. Physical phenotype is obviously a candidate, but we propose that body-odour could, and does, serve as an added effective cue. In other words, we propose that humans are smelling themselves also as part of a matching-mechanism in social interaction. Indeed, we propose that it is the loss of this ability that may underlie part of the social difficulties associated with congenital anosmia [53,54].

Finally, we think that humans sniff themselves also to obtain information on their transient physiological and emotional state. Body-odour reflects general arousal [55] and may specifically reflect emotions ranging from fear [56,57] to happiness [58] and beyond. Thus, self-sniffing may be part of a general mechanism for emotional homeostasis [59]. In this, we may be proposing a new arena for contrasting of the James–Lange/Cannon–Bard/Schacter–Singer debate [60]. Do we sniff ourselves, smell fear and then become afraid? Or experience fear and smell it on ourselves at the same time? Or some combination of these? This too is an area for future investigation.

## 10. Why has olfactory self-sniffing received such scant attention?

So humans are sniffing the environment [61], sniffing others, and sniffing themselves (see [8,9,12], and current self-report survey). This behaviour is troubling to people when they become aware of it (e.g. [‘hand sniffing’ children] Google search), yet it has near zero traction in medical and psychological research. Why? We think that self-sniffing has been disregarded for reasons mostly common with the general disregard of human olfaction in twentieth–twenty-first century thinking. Much has been written on this (e.g. [62–65]), and from this, we will extract three primary reasons.

First, sniffing conspecifics is viewed as animalistic behaviour. Although we have come a long way since ‘we will hope it is not true. But if it is, let us pray that it may not become generally known', scientists nevertheless remain reluctant to consider behavioural parallels between humans and other animals such as meerkats, goats and donkeys, particularly in behaviours related to mating. Sniffing others in this respect is considered ‘undignified' [63].

Second, odours are associated with filth, and a short path from moral filth [31]. This relatively new (in an evolutionary perspective) and current status of odour is well illustrated by the following example: in 1796, Napoleon famously wrote to Josephine asking her not to bathe before his eminent return home, supposedly so that he would take increased pleasure in her body-odour (attributed to [66]). Today, only slightly more than 200 years later, most Westerners would literally cringe at such a thought. Given that human sensory physiology has not dramatically changed over the past 200 years, it is a change in attitude that underlies this shift. This shift in attitude may be attributed to the uncovering of the link between germs and disease [31]. This shifted odours from the position of agents that fight infection (up to about the time of Napoleon) to the position of an agents that signal infection (in our times). Bodily odours became a sign of something unclean and disease prone, and therefore their sampling may also be viewed as a negative action.

Finally, there is no escaping the fact that human olfaction is still widely perceived as dull and unimportant, and as such, it is unsurprising that its mechanisms of acquisition within social settings have been largely overlooked. Despite some recent efforts of review manuscripts highlighting the acuity and importance of human olfaction [65,67,68], all of these authors are facing a remarkable power: Sigmund Freud (figure 10*d*). No single person has had a greater impact on current attitudes towards human psychology and behaviour [69]. Freud, especially in his later writing [64], and aligning with Havelock Ellis in this respect [70], denigrated the behavioural significance of human olfaction, particularly in healthy sexual behaviour, where he saw no role for it. Freud argued that in the evolutionary transition to bipedalism, olfactory interests shifted away from the sexual organs (that were no longer directly in front of the nose), and that any such current interest therefore reflects a regression into animalism, or in other words, a pathological state [64]. An interesting analysis went so far as to suggest that Freud had a ‘nasal complex' [71]. In this analysis, Freud's nasal complex was attributed to the history of the relationship between Freud and Wilhelm Fliess. Fliess, an ENT physician, was a close and influential friend of Freud [72]. Much like the authors of this manuscript, Fliess thought that many aspects of human behaviour, and particularly sexual behaviour, could be attributed to olfaction and the nose [73–75]. Fliess in fact conducted two lots of surgery on Freud's nose in order to address various conditions [71]. Freud also called on Fliess to assist in treating the ‘sexual neuroticism’ of his patient Emma Eckstein. Fliess proceeded to treat this condition by obvious means: surgery on her nose (…) [75]. Moreover, Fliess proceeded to forget a long string of gauze within Eckstein's nose, the later surgical removal of which was purportedly a profoundly traumatic event for Freud (and no doubt for her) [71]. According to Howes, this traumatic event was at the heart of the ensuing fallout between Freud and Fliess, and the source of Freud's ‘nasal complex’. Whatever the reason was, Freud indeed made no allowance for olfaction in his accounts of healthy human behaviour, sexual or other. Given the significant impact of Freudian psychology on current views of human behaviour, if olfaction is indeed not seen as a factor in healthy ongoing human behaviour, it is unsurprising that olfactory sampling behaviour remains unnoticed.

## 11. Limitations and future research

Our account of human behaviour still faces at least one major challenge or limitation: whereas we see the majority of face-touching behaviour as instances of hand-sniffing, others may argue that these are mere incidental touches of the faces, perhaps a form of displacement behaviour [13], with no link to olfaction or social chemosignalling. The current empirical arguments we present in this respect are that people double nasal airflow when they bring their hand to their nose (figure 1*b*), and that olfactory tainting can increase or decrease face-touching in a predicted fashion (figure 9*a*). Although both of these are arguments for an olfactory behaviour, they both come from one study out of one laboratory––ours. Thus, added verifications, from us and others, as to the olfactory origins of face-touching are absolutely necessary for solidification of our hypothesis. One line of verification depends on technical advance: we desperately need a method to precisely measure nasal airflow from a distance. Our current measurements depend on placing a nasal cannula at the nose, and this in itself may alter behaviour. Our laboratory has experimented to this end with forward-looking infra-red (FLIR) cameras, but we have failed to reach satisfactory results, and this remains a major challenge. A second line of potential hypothesis testing is to measure face-touching in individuals without a sense of smell. Notably, whether anosmia necessarily implies social anosmia also still remains a critical open question in the field [76,77], but if it does, then face-touching behaviour should be altered in anosmia, particularly congenital anosmia. Finally, if one accepts our thesis on hand-sniffing as a significant component of human behaviour, this opens a field of questions in social sniffing: What are the neuronal mechanisms of this behaviour? How is it related to different ongoing behaviours? Is self-smelling altered in particular pathologies? And finally, can we harness this behaviour towards therapy? Although we again stress the need for additional verification studies on the olfactory origins of face-touching, we end in stating that we think human self-smelling is a significant yet overlooked aspect of human behaviour.

## Supplementary Material

Video S1: Humans spontaneously sniff their own hands

## Supplementary Material

Video S2: Humans sniff their hands after handshake

## Supplementary Material

File S1: Online questionnaire

## Supplementary Material

File S2: raw data

## Supplementary Material

Supplementary Figure S1

## Data accessibility

All the raw data are presented in an electronic supplementary material file.

## References

[1] Mainland J, Sobel N 2005 The sniff is part of the olfactory percept. Chem. Senses 31, 181–196. (10.1093/chemse/bjj012) 16339268

[2] Wilson RI 2008 Neural and behavioral mechanisms of olfactory perception. Curr. Opin. Neurobiol. 18, 408–412. (10.1016/j.conb.2008.08.015) 18809492 PMC2596880

[3] Schoenfeld TA, Cleland TA 2005 Anatomical contributions to odorant sampling and representation in rodents: zoning in on sniffing behavior. Chem. Senses 31, 131–144. (10.1093/chemse/bjj015) 16339266

[4] Kepecs A, Uchida N, Mainen ZF 2005 The sniff as a unit of olfactory processing. Chem. Senses 31, 167–179. (10.1093/chemse/bjj016) 16339265

[5] Wachowiak M 2011 All in a sniff: olfaction as a model for active sensing. Neuron 71, 962–973. (10.1016/j.neuron.2011.08.030) 21943596 PMC3237116

[6] Dimond S, Harries R 1984 Face touching in monkeys, apes and man: evolutionary origins and cerebral asymmetry. Neuropsychologia 22, 227–233. (10.1016/0028-3932(84)90065-4) 6427652

[7] Suarez SD, Gallup GG Jr 1986 Face touching in primates: a closer look. Neuropsychologia 24, 597–600. (10.1016/0028-3932(86)90105-3) 3774145

[8] Nicas M, Best D 2008 A study quantifying the hand-to-face contact rate and its potential application to predicting respiratory tract infection. J. Occup. Environ. Hyg. 5, 347–352. (10.1080/15459620802003896) 18357546 PMC7196690

[9] Kwok YLA, Gralton J, McLaws M-L 2015 Face touching: a frequent habit that has implications for hand hygiene. Am. J. Infect. Control 43, 112–114. (10.1016/j.ajic.2014.10.015) 25637115 PMC7115329

[10] Elder NC, Sawyer W, Pallerla H, Khaja S, Blacker M 2014 Hand hygiene and face touching in family medicine offices: a Cincinnati Area Research and Improvement Group (CARInG) network study. J. Am. Board Family Med. 27, 339–346. (10.3122/jabfm.2014.03.130242) 24808112

[11] Rabie T, Curtis V 2006 Handwashing and risk of respiratory infections: a quantitative systematic review. Trop. Med. Int. Health 11, 258–267. (10.1111/j.1365-3156.2006.01568.x) 16553905 PMC7169664

[12] Frumin I et al. 2015 A social chemosignaling function for human handshaking. eLife 4, e05154 (10.7554/eLife.05154) PMC4345842 25732039

[13] Troisi A 2002 Displacement activities as a behavioral measure of stress in nonhuman primates and human subjects. Stress 5, 47–54. (10.1080/102538902900012378) 12171766

[14] Arzi A et al. In press. Olfactory sniffing signals consciousness in vegetative patients. Nature. 10.1038/s41586-020-2245-5 32461641

[15] Milinski M, Wedekind C 2001 Evidence for MHC-correlated perfume preferences in humans. Behav. Ecol. 12, 140–149. (10.1093/beheco/12.2.140)

[16] Darling DA 1957 The Kolmogorov-Smirnov, Cramer-von Mises tests. Ann. Math. Stat. 28, 823–838. (10.1214/aoms/1177706788)

[17] Van Gelder MM, Bretveld RW, Roeleveld N 2010 Web-based questionnaires: the future in epidemiology? Am. J. Epidemiol. 172, 1292–1298. (10.1093/aje/kwq291) 20880962

[18] Classen C 1992 The odor of the other: olfactory symbolism and cultural categories. Ethos 20, 133–166. (10.1525/eth.1992.20.2.02a00010)

[19] Perry S 2011 Social traditions and social learning in capuchin monkeys (*Cebus*). Phil. Trans. R. Soc. B 366, 988–996. (10.1098/rstb.2010.0317) 21357221 PMC3049088

[20] Firth R 1972 Verbal and bodily rituals of greeting and parting. Interpret. Ritual 1972, 1–38.

[21] Schiffrin D 1974 Handwork as ceremony: the case of the handshake. Semiotica 12, 189–202. (10.1515/semi.1974.12.3.189)

[22] Endevelt-Shapira Y et al. 2018 Altered responses to social chemosignals in autism spectrum disorder. Nat. Neurosci. 21, 111 (10.1038/s41593-017-0024-x) 29180748

[23] Dinstein I, Heeger DJ, Lorenzi L, Minshew NJ, Malach R, Behrmann M 2012 Unreliable evoked responses in autism. Neuron 75, 981–991. (10.1016/j.neuron.2012.07.026) 22998867 PMC3457023

[24] Sacks O 2012 An anthropologist on Mars: seven paradoxical tales. New York, NY: Vintage. 10.4103/0028-3886.258001 31085904

[25] Pause BM, Krauel K, Sojka B, Ferstl R 1998 Body odor evoked potentials: a new method to study the chemosensory perception of self and non-self in humans. Genetica 104, 285–294. (10.1023/A:1026462701154) 10386395

[26] Lundström JN, Boyle JA, Zatorre RJ, Jones-Gotman M 2007 Functional neuronal processing of body odors differs from that of similar common odors. Cereb. Cortex 18, 1466–1474. (10.1093/cercor/bhm178) 17934190

[27] Lundström JN, Boyle JA, Zatorre RJ, Jones-Gotman M 2009 The neuronal substrates of human olfactory based kin recognition. Hum. Brain Mapp. 30, 2571–2580. (10.1002/hbm.20686) 19067327 PMC6870682

[28] McClintock MK 2002 Pheromones, odors, and vasanas: the neuroendocrinology of social chemosignals in humans and animals. In Hormones, brain and behavior (eds Pfaff DW, Arnold AP, Etgen AM, Fahrbach SE, Rubin RT), pp. 797–870. Amsterdam, The Netherlands: Elsevier.

[29] Luebke KT, Pause BM 2015 Always follow your nose: the functional significance of social chemosignals in human reproduction and survival. Horm. Behav. 68, 134–144. (10.1016/j.yhbeh.2014.10.001) 25637403

[30] Semin GR, de Groot JH 2013 The chemical bases of human sociality. Trends Cogn. Sci. 17, 427–429. (10.1016/j.tics.2013.05.008) 23796879

[31] Soo ML, Stevenson RJ 2007 The moralisation of body odor. Mankind Q. 47, 25–56.

[32] Olsson MJ et al. 2014 The scent of disease: human body odor contains an early chemosensory cue of sickness. Psychol. Sci. 25, 817–823. (10.1177/0956797613515681) 24452606

[33] Gergely G, Watson JS 1996 The social biofeedback theory of parental affect-mirroring: the development of emotional self-awareness and self-control in infancy. Int. J. Psycho-Anal. 77, 1181–1212. 9119582

[34] Kernberg PF, Buhl-Nielsen BC, Normandin LC 2006 Beyond the reflection: the role of the mirror paradigm in clinical practice. New York, NY: Other Press.

[35] Enoch JM 2006 History of mirrors dating back 8000 years. Optom. Vis. Sci. 83, 775–781. (10.1097/01.opx.0000237925.65901.c0) 17041324

[36] Well T 2018 The clear mirror meditation practice guide.

[37] Übel S, Wabnegger A, Arendasy M, Zorjan S, Schienle A 2017 Affective evaluation of one's own and others’ body odor: the role of disgust proneness. Perception 46, 1427–1433. (10.1177/0301006617721120) 28705102

[38] Platek SM, Burch RL, Gallup GG Jr 2001 Sex differences in olfactory self-recognition. Physiol. Behav. 73, 635–640. (10.1016/S0031-9384(01)00539-X) 11495669

[39] McBurney DH, Levine JM, Cavanaugh PH 1976 Psychophysical and social ratings of human body odor. Pers. Soc. Psychol. Bull. 3, 135–138. (10.1177/014616727600300126)

[40] Hold B, Schleidt M 1977 The importance of human odour in non-verbal communication. Z. Tierpsychol. 43, 225–238. (10.1111/j.1439-0310.1977.tb00072.x) 868320

[41] Russell MJ 1976 Human olfactory communication. Nature 260, 520–522. (10.1038/260520a0) 1264204

[42] Schleidt M 1980 Personal odor and nonverbal communication. Ethol. Sociobiol. 1, 225–231. (10.1016/0162-3095(80)90009-6)

[43] Olsson SB, Barnard J, Turri L 2006 Olfaction and identification of unrelated individuals: examination of the mysteries of human odor recognition. J. Chem. Ecol. 32, 1635 (10.1007/s10886-006-9098-8) 16900423

[44] Mallet P, Schaal B 1998 Rating and recognition of peers' personal odors by 9-year-old children: an exploratory study. J. Gen. Psychol. 125, 47–64. (10.1080/00221309809595576) 9580975

[45] Salomon R 2017 The assembly of the self from sensory and motor foundations. Soc. Cogn. 35, 87–106. (10.1521/soco.2017.35.2.87)

[46] Well T 2018 Why is seeing your own reflection so important? *Psychol. Today.* See https://www.psychologytoday.com/us/blog/the-clarity/201808/why-is-seeing-your-own-reflection-so-important.

[47] Parker L 2015 This is what happened when I didn't look in a mirror for a week. New York, NY: BuzzFeed See https://www.buzzfeed.com/laraparker/11-things-i-learned-after-not-looking-in-a-mirror-for-a-week.

[48] Jacob S, McClintock MK, Zelano B, Ober C 2002 Paternally inherited HLA alleles are associated with women's choice of male odor. Nat. Genet. 30, 175 (10.1038/ng830) 11799397

[49] Wedekind C, Füri S 1997 Body odour preferences in men and women: do they aim for specific MHC combinations or simply heterozygosity? Proc. R. Soc. Lond. B 264, 1471–1479. (10.1098/rspb.1997.0204) PMC1688704 9364787

[50] Wedekind C, Seebeck T, Bettens F, Paepke AJ 1995 MHC-dependent mate preferences in humans. Proc. R. Soc. Lond. B 260, 245–249. (10.1098/rspb.1995.0087) 7630893

[51] Fowler JH, Settle JE, Christakis NA 2011 Correlated genotypes in friendship networks. Proc. Natl Acad. Sci. USA 108, 1993–1997. (10.1073/pnas.1011687108) 21245293 PMC3033315

[52] Christakis NA, Fowler JH 2014 Friendship and natural selection. Proc. Natl Acad. Sci. USA 111, 10 796–10 801. (10.1073/pnas.1400825111) PMC4113922 25024208

[53] Croy I, Negoias S, Novakova L, Landis BN, Hummel T 2012 Learning about the functions of the olfactory system from people without a sense of smell. PLoS ONE 7, e33365 (10.1371/journal.pone.0033365) 22457756 PMC3310072

[54] Croy I, Bojanowski V, Hummel T 2013 Men without a sense of smell exhibit a strongly reduced number of sexual relationships, women exhibit reduced partnership security–a reanalysis of previously published data. Biol. Psychol. 92, 292–294. (10.1016/j.biopsycho.2012.11.008) 23178326

[55] Adolph D, Schlösser S, Hawighorst M, Pause BM 2010 Chemosensory signals of competition increase the skin conductance response in humans. Physiol. Behav. 101, 666–671. (10.1016/j.physbeh.2010.08.004) 20708023

[56] Zhou W, Chen D 2009 Fear-related chemosignals modulate recognition of fear in ambiguous facial expressions. Psychol. Sci. 20, 177–183. (10.1111/j.1467-9280.2009.02263.x) 19170944

[57] Chen D, Katdare A, Lucas N 2006 Chemosignals of fear enhance cognitive performance in humans. Chem. Senses 31, 415–423. (10.1093/chemse/bjj046) 16527869

[58] de Groot JH, Smeets MA, Rowson MJ, Bulsing PJ, Blonk CG, Wilkinson JE, Semin GR 2015 A sniff of happiness. Psychol. Sci. 26, 684–700. (10.1177/0956797614566318) 25870406

[59] Lewis MD 2005 Bridging emotion theory and neurobiology through dynamic systems modeling. Behav. Brain Sci. 28, 169–194. (10.1017/S0140525X0500004X) 16201458

[60] Gazzaniga M, Ivry RB 2013 Cognitive neuroscience: the biology of the mind, 4th int. student edn New York, NY: WW Norton.

[61] Arzi A, Shedlesky L, Secundo L, Sobel N 2014 Mirror sniffing: humans mimic olfactory sampling behavior. Chem. Senses 39, 277–281. (10.1093/chemse/bjt113) 24457159 PMC3982906

[62] Stoddart DM 1990 The scented ape: the biology and culture of human odour. Cambridge, UK: Cambridge University Press.

[63] Corbin A 1986 The foul and the fragrant: odor and the French social imagination. Cambridge, MA: Harvard University Press.

[64] Harrington A, Rosario V 1992 Olfaction and the primitive: nineteenth-century medical thinking on olfaction. In Science of olfaction (eds Serby MJ, Chobor KL), pp. 3–27. New York, NY: Springer.

[65] McGann JP 2017 Poor human olfaction is a 19th-century myth. Science 356, eaam7263 (10.1126/science.aam7263) 28495701 PMC5512720

[66] de Tourtier-Bonazzi C, Tulard J 1981 Napoléon. Lettres d'amour à Joséphine [Napoleon. Love letters to Josephine]. Paris, France: Fayard [In French.]

[67] Stevenson RJ 2009 An initial evaluation of the functions of human olfaction. Chem. Senses 35, 3–20. (10.1093/chemse/bjp083) 19942579

[68] Shepherd GM 2004 The human sense of smell: are we better than we think? PLoS Biol. 2, e146 (10.1371/journal.pbio.0020146) 15138509 PMC406401

[69] Haggbloom SJ et al. 2002 The 100 most eminent psychologists of the 20th century. Rev. Gen. Psychol. 6, 139–152. (10.1037/1089-2680.6.2.139)

[70] Kalogerakis MG 1963 The role of olfaction in sexual development. Psychosom. Med. 25, 420–432. (10.1097/00006842-196309000-00002) 14050424

[71] Howes D 2010 Sensual relations: engaging the senses in culture and social theory. Ann Arbor, MI: University of Michigan Press.

[72] Gay P 1998 Freud: a life for our time. New York, NY: WW Norton & Company.

[73] Fliess W 1893 Die nasale reflexneurose [The nasal reflexneurosis] Wiesbaden, Germany: JF Bergman [In German.]

[74] Young AR 2002 Freud's friend Fliess. J. Laryngol. Otol. 116, 992–995. (10.1258/002221502761698702) 12537609

[75] Zucker A, Wiegand D 1988 Freud, Fliess, and the nasogenital reflex: did a look into the nose let us see the mind? Otolaryngol. Head Neck Surg. 98, 319–322. (10.1177/019459988809800409) 3132686

[76] Frasnelli J, Lundström JN, Boyle JA, Katsarkas A, Jones-Gotman M 2011 The vomeronasal organ is not involved in the perception of endogenous odors. Hum. Brain Mapp. 32, 450–460. (10.1002/hbm.21035) 20578170 PMC3607301

[77] Savic I, Hedén-Blomqvist E, Berglund H 2009 Pheromone signal transduction in humans: what can be learned from olfactory loss. Hum. Brain Mapp. 30, 3057–3065. (10.1002/hbm.20727) 19235878 PMC6870699

