# Supplementary material for: Are humans constantly but subconsciously smelling themselves?
Source: Philos Trans R Soc Lond B Biol Sci. 2020 Apr 20;375(1800):20190372. doi: 10.1098/rstb.2019.0372 (PMC7209943; doi:10.1098/rstb.2019.0372)
Supplement: File S1: Online questionnaire [file rstb20190372supp3.pdf]

SNIFF SOMEONE YOU LOVE

Dear Participant

We are asking for your help in this online questionnaire where we want to quantify the prevalence of olfactory sampling and self-sampling behavior. Such behavior is oddly treated as either taboo at one end, or a source of humor on the other, yet we genuinely want to obtain estimates of its prevalence.

You can answer all or only part of the questions.

After you submit your answers, you will see the average answer to each question

The questionnaire is anonymous

1. **Age**

   ____

2. **Gender**

   Mark only one oval.

   - Female
   - Male
   - Prefer not to say
   - Other: ______________________________

3. **Country of residence**

   ____

4. *Check all that apply.*

	N/A	Never	Rarely	Occasionally	Often
Do you ever sniff/smell your romantic partner?	☐	☐	☐	☐	☐
Do you ever sniff/smell your children?	☐	☐	☐	☐	☐
Do you ever sniff/smell strangers?	☐	☐	☐	☐	☐
Do you ever sniff/smell your own hands	☐	☐	☐	☐	☐
Do you ever sniff/smell your own underarm?	☐	☐	☐	☐	☐
Do you ever place your hands in your underarms and then smell them?	☐	☐	☐	☐	☐
Do you ever place your hands in your crotch and then smell them?	☐	☐	☐	☐	☐
Do you ever smell your own socks/shoes after you wore them?	☐	☐	☐	☐	☐
Do you ever smell your own shirt after you wore it?	☐	☐	☐	☐	☐
Do you ever smell your own underpants after you wore them?	☐	☐	☐	☐	☐

5. *Check all that apply.*

	N/A	Yes	No
If you have children (even if they are now older) was there a phase or age where they often sniffed their own hands?	☐	☐	☐

6. **If you answered yes to the above, can you estimate the age at which this behavior was at its peak?**

Mark only one oval.

- ◯ 2
- ◯ 3
- ◯ 4
- ◯ 5
- ◯ 6
- ◯ 7
- ◯ 8
- ◯ 9
- ◯ 10
- ◯ 11
- ◯ 12
- ◯ 13
- ◯ 14
- ◯ 15
- ◯ 16
- ◯ 17
- ◯ 18

Powered by
Google Forms
