# Supplementary material for: Are humans constantly but subconsciously smelling themselves?
Source: Philos Trans R Soc Lond B Biol Sci. 2020 Apr 20;375(1800):20190372. doi: 10.1098/rstb.2019.0372 (PMC7209943; doi:10.1098/rstb.2019.0372)
Supplement: Supplementary Figure S1 [file rstb20190372supp5.pdf]

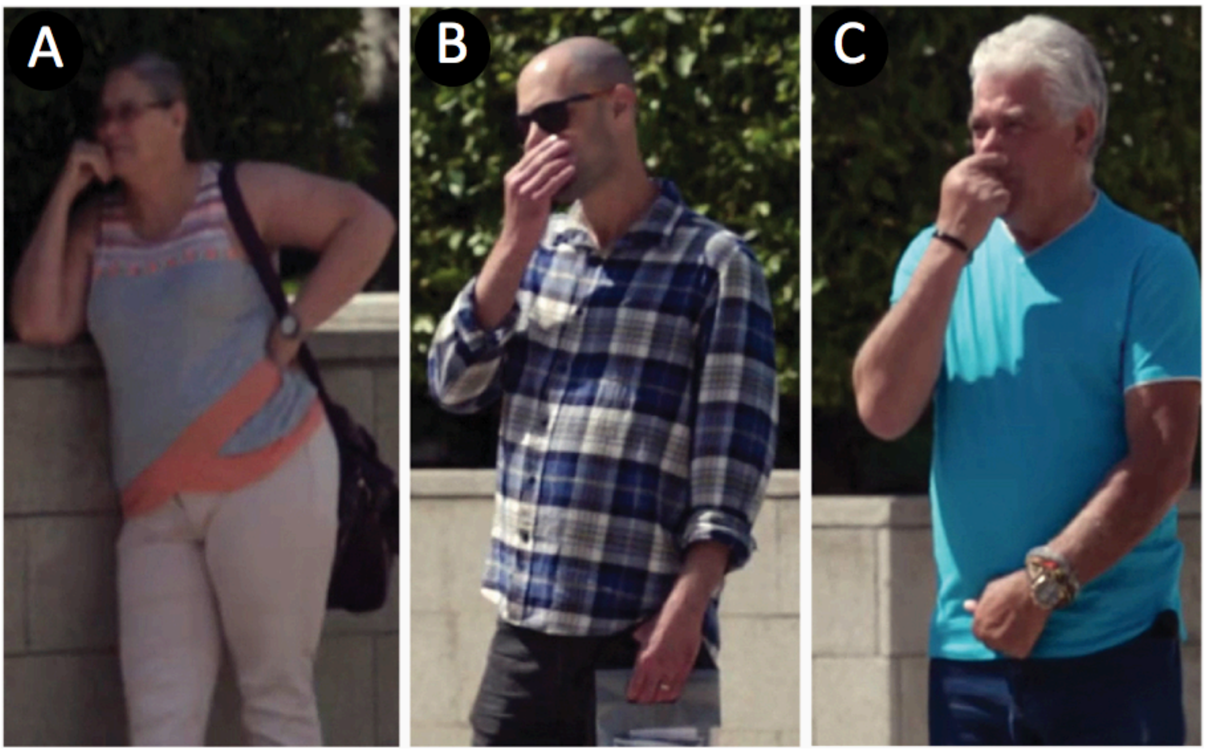

Supplementary Figure 1: Face-touching after handshake

A-C. Face-touching following handshake in an educational television show rendition. Note the contrast between the implicit behaviour in A versus the explicit behaviour in B and C (see Supplementary Video 2, https://youtu.be/0WL0zdEhzUM).

Amusingly, the handshake effect has been "replicated" in live recreations on several popularized science television shows around the world. We use one for which we could receive rights to append as an online video, in order to highlight the nuances of these observations (Supplementary Video 2, https://youtu.be/0WL0zdEhzUM). This show used a modified rendition whereby they approached several people on the street and told them they were selected for a televised trivia challenge. They lined them up (about 4 or 5 at a time), shook their hands, then took one away for trivia questioning, while covertly filming the others. Whereas our manuscript reports about 50% incidence of hand-sniffing after handshake, in their first take they reported that 2 out of 3 observers hand-sniffed., i.e., ~66% incidence. However, we note that the person they did not count, in fact also hand-sniffed by our criteria (Figure 6C), yet this was less overt than the other two (Figure 6D, 6E). Thus, this popularized rendition had 100% incidence in this take (and 50% in an ensuing take). This nuance in observational criteria serves to highlight that we think most hand-sniffing is in fact of the implicit type, and not the very explicit.
